# Mental Health Symptoms, Binge Drinking, and the Experience of Abuse During the COVID-19 Lockdown in Mexico

**DOI:** 10.3389/fpubh.2021.656036

**Published:** 2021-07-22

**Authors:** Silvia Morales Chainé, Alejandra López Montoya, Alejandro Bosch Maldonado, Ana Beristain Aguirre, Rebeca Robles García, Carlos Rodrigo Garibay Rubio, Claudia Iveth Astudillo García, Isaura Angélica Lira Chávez, María Gudelia Rangel Gómez

**Affiliations:** ^1^Universidad Nacional Autónoma de México, Mexico City, Mexico; ^2^Instituto Nacional de Psiquiatría “Ramón de la Fuente Muñíz”, Mexico City, Mexico; ^3^Secretaría de Gestión Integral de Riesgos y Protección Civil de la Ciudad de México, Mexico City, Mexico; ^4^Servicios de Atención Psiquiátrica de la Secretaría de Salud, Mexico City, Mexico; ^5^Sección mexicana de la Comisión de Salud Fronteriza México-Estados Unidos, Mexico City, Mexico; ^6^El Colegio de la Frontera Norte, Mexico City, Mexico

**Keywords:** mental health symptoms, stress, anxiety, binge drinking, the experience of abuse, COVID-19

## Abstract

**Background:** The health crisis associated with the COVID-19 pandemic is causally linked to negative mental health symptoms in the same way as other diseases such as Ebola.

**Objective:** The purpose of this paper is to describe the relationship between mental health symptoms, binge drinking, and the experience of abuse during the COVID-19 lockdown.

**Method:** We surveyed 9,361 participants, all Mexican, with an average age of 33 years old (*SD* = 10.86). In this group of people, we found out that 59% were single (5,523), 71% were women (6,693). Forty-six percentage were complying with lockdown procedures (4,286), 50% were partially complying (4,682), and 4% were not complying at all (393). The invitation to participate was open from April 24th to April 30th during the second stage of the pandemic in Mexico, in 2020, characterized by voluntary complete lockdown staying at home. Thus, we used a cross-sectional online survey design to assess mental health risk factors related to the COVID-19 pandemic. The survey was available on a WebApp designed by Linux^®^, PHP^®^, HTML^®^, CSS^®^, and JavaScript^®^. We calculated descriptive and inferential analysis to describe the mental health average distribution as a function of the lockdown, binge drinking, and experience of abuse. To calculate the reliability and validation of the subscales, we used Cronbach's Alpha and Factor Loading. We run the confirmatory factor loading analysis, and we described the relationship between each latent variable and its item factor load, obtained through structural modeling equations, derived from 179 iterations and 207 parameters (*t*_[1,171]_ = 28,079.418, *p* < 0.001). We got a CFI of 0.947, a TLC of 0.940, an RMSEA of 0.049 (0.049–0.050), and an SRMR of 0.048.

**Findings:** The results indicated that reported attitudes such as avoidance, sadness, withdrawal, anger, and anxiety were associated with acute stress, which was linked to an anxiety condition caused by uncertainty about achieving or maintaining overall good health.

**Discussion and Prospects:** People in lockdown mentioned a sudden increase in alcohol consumption. They lived episodes of physical and emotional abuse, in contrast with those who stated that they did not go into lockdown or consume alcohol, or experienced abuse.

**Limitations:** Further studies should diagnose mental health conditions as part of the impact of COVID-19, ensure their follow-up, and assess the effect of providing remote psychological care. There is a need to explore methods to curb the increase in the number of people affected by post-traumatic stress disorder.

## Background

The risk of becoming infected by the SARS-CoV-2, which causes COVID-19, soared in late 2019 in Hubei, China. The virus gradually spread across the globe. By May 2020, 3,516,240 people had been diagnosed with the virus, of which 249,092 died and 1,086,620 recovered (1). The disease, which mainly affects the respiratory system, has a higher mortality rate than seasonal flu. By May 2020, Mexico had 23,471 cases of COVID-19, 12,664 suspected cases, and 59,704 had tested negative. Also, 13,447 people had recovered, and 2,154 had died (2). The death rate was 9.2 per 100,000 population.

The Lancet (3) subsequently established that most countries experience some disruption of mental, neurological, and substance use services, impacting community-based programs. According to this publication, 17% of 116 counties had earmarked additional funding for mental health and psychological support related to the COVID-19 pandemic to improve the world population's mental health and well-being.

The Lancet (3) also reported that, historically, 33–42% of those admitted to hospital for severe acute respiratory syndrome or Middle East respiratory syndrome during Ebola outbreaks suffered from depressed mood, anxiety, impaired memory, and insomnia, which continued beyond recovery. It appears that in the case of COVID-19, non-pharmaceutical interventions have led to the abuse of substances, particularly alcohol. At the same time, the attendant health symptoms are more likely to affect lower-income countries. In a systematic review and meta-analysis, Rogers et al. (4) assessed the psychiatric and neuropsychiatric presentations of SARS, MERS, and COVID-19. They selected studies with data on individuals with suspected or laboratory-confirmed coronavirus infection. They found psychiatric signs or symptoms, symptom severity, diagnoses based on ICD-10, DSM-IV, quality of life, and employment across illness stages: acute vs. post-illness. The authors selected 65 peer-reviewed studies, seven preprints from 1,963 reviews, and 87 preprints. The number of coronavirus cases in the studies included was 3,559, with subjects aged between 12.2 years old and 68 years old, while the follow-up time for the post-illness studies varied between 60 days and 12 years. The findings indicated that people suffered from confusion, depression, anxiety, impaired memory, and insomnia. These symptoms had been associated with acute illness and common symptoms among patients admitted to the hospital for SARS or MERS. At the post-illness stage, the authors found depression, insomnia, anxiety, irritability, memory impairment, fatigue, traumatic memories, and sleep disorder, and post-traumatic stress disorder in the study subjects.

Given this context and the genuine threat of death, we must direct a series of procedures to prevent the spread of COVID-19 while maintaining a certain level of mental health. The first wave of consequences includes acute stress and emotional situations. Stress is an adaptative response; preparing us for critical situations. However, we defined stress as physiological, motor, behavioral, cognitive, and emotional reactions, characterized by a *fight or flight* response or shut down, depending on the individual (5, 6). As a consequence of the level of risk, people will tend to feel overwhelmed both psychologically and emotionally, reporting a loss of appetite, fatigue, insomnia, irritability, attention deficit, and fear. These symptoms can lead to binge drinking and violent outbursts (7).

Regarding the hazards of contracting COVID-19, Li et al. (7) have assessed a series of mental health risks using a WebApp, measuring them through a scale of vicarious trauma. They surveyed their participants during the first week of lockdown in China. Findings included a persistent vicarious trauma level among respondents: specific physiological, emotional, behavioral, and cognitive responses based on the context, their fear of contagion, and anxiety caused by lockdown itself.

According to Selye (8) and De Camargo (9), people, in general, can respond to a traumatic or stressful event through a general adaptation syndrome, composed of an awake phase, a resistance phase, and an exhaustion phase. This last phase is when the following reactions occur: insomnia, fatigue, lack of concentration, cardiovascular and metabolic reactions, endocrine responses, emotional problems, gastrointestinal issues, and vascular events, among others (5, 6, 10, 11).

It is, therefore, evident that people can display a wide array of negative emotions (rejection, anxiety; 8) and a negative cognitive evaluation of the COVID-19 pandemic (12). Li et al. (12) assessed negative and cognitive emotions (anxiety, depression, outrage and unhappiness, social risk assessment, and satisfaction) during the first 3 weeks of lockdown by screening 17,865 individuals, 75% of whom were girls or women aged between eight and 56 (*M* = *33)*. After conducting an online survey, the authors observed a spike in the negative emotions (anxiety, depression, and outrage). There was a decrease in positive emotions (happiness) that correlated with being at risk of contagion. Regarding cognitive indicators, negative attitudes increased, and life satisfaction levels decreased.

Another study conducted in China (13) showed that 54% of those under lockdown experienced a high, severe psychological impact, with 16.5% reporting moderate to severe depression symptoms, 29% signs of anxiety, and 8% mild to extreme, higher than normal stress levels. These authors also found that people are more fearful than they were during the SARS pandemic in 2008. Exposure to the risk of contagion creates high anxiety levels due to possible outcomes, such as severe illness, death, hopelessness, misery, or guilt linked to the infection of other people and the use of alcohol and violence.

In Mexico, Morales-Chainé et al. (14) evaluated stress levels, emotions, and negative thoughts during the second stage of the COVID-19 pandemic, when lockdown began (March 2020), using sociodemographic variables. The authors surveyed 1,906 individuals (*M* = *29; SD* = *9.53)* between the ages of 14 and 69, 65% of whom reported complying with lockdown procedures. Participants answered the Screening of Mental Health during Emergencies Survey (via a WebApp available for Android^®^, iOS^®^, Symbian^®^, Windows^®^, WebOS^®^). The authors concluded that people in lockdown were more prone to develop negative emotions (lack of interest, self-harm intentions, insomnia, and difficulty concentrating).

Recently, Morales-Chainé et al. (15) described mental health symptoms associated with a diagnosis of COVID-19, the death of people closes to one, or a previous depression diagnosis. They surveyed 15,335 Mexicans with a mean age of 26 (*SD* = 11.95), 44% of whom were under lockdown (6,769), using the WebApp Questionnaire on Mental Health Screening in COVID-19 (Alpha = 0.96; 62% explained variance). Results indicated an association between avoidance symptoms, acute stress, generalized anxiety, sadness, and anger in people during the COVID-19 pandemic. They found mental health symptoms related to self-isolation, a COVID-19 diagnosis, and reported comorbidity due to physical illness or depression.

In this respect, mental health conditions associated with the risk of being infected with COVID-19 also include thought avoidance processes to prevent the triggering. The COVID-19 pandemic poses a constant emotional threat placing vulnerable individuals in a continuous state of alert with their emotional responses. Given the duration of the pandemic, PTSD situations could affect an ever-increasing number of individuals.

We argue that both the risk of becoming infected with COVID-19 and the living conditions imposed by lockdowns are associated with mental health threats, specifically those related to acute stress, negative thoughts, emotions, and anxiety. All these symptoms can develop into acute stress. Other risks associated with lockdowns include binge drinking and the experience of abuse, which increase the odds of developing PTSD (16). Accordingly, the purpose of this paper was to describe the relationship between mental health symptoms, binge-drinking, and the experience of abuse during the COVID-19 lockdown. The research undertaken to write this paper enabled us to identify a set of variables regarding acute stress, avoidance-sadness, distancing-anger, generalized anxiety, health-related anxiety, binge-drinking, and the experience of abuse in Mexico.

## Method

### Design

We used a cross-sectional online survey design to assess mental health risk factors related to the COVID-19 pandemic. At the beginning of the second stage of the COVID-19 pandemic in Mexico, when the lockdown was imposed (April 24–30, 2020), we surveyed participants.

We invited the general population to participate through a public announcement on the official Health Ministry website and the institutional website of the leading public university in Mexico. They had to log into the system with their Unique Population Registry Code, their university account number, or their email to ensure traceability of participation. The invitation to participate was open from April 24th to April 30th during the second stage of the pandemic in Mexico, in 2020, characterized by a complete voluntary lockdown staying at home. Stage one consisted of keeping a healthy distance from people and taking care yet without going into lockdown. The Mexican Undersecretary of Health declared the second phase of the pandemic on March 23rd, 2020, describing it as the community spread stage in Mexico. During the week we surveyed the participants, lockdown had lasted between 30 and 37 days.

### Participants

To conduct our research, we surveyed 9,361 participants (see Table 1), with an average age of 33 (*SD* = 10.86, ranging from 9 to 86). Twenty participants were pre-adolescents under 15, so we asked them to provide their parents' email to authorize their participation [According to the ESOMAR World Research Codes & Guidelines interviewing children and young people (17) and establishing caring children under 14 years]. Fifty-nine percentage of respondents were single, and 71% female (6,693).

**Table 1:** Number of respondents with lockdown status during the COVID-19 pandemic, gender, employment status, and clinical characteristics, including sex.

	**Lockdown/Sex**															
	**In lockdown**				**In partial lockdown**				**Not in lockdown**				**Total**			
**Sex**	**Women**		**Men**		**Women**		**Men**		**Women**		**Men**		**Women**		**Men**	
**Sex** ***n (%)***	**3,198 (75%)**		**1,088 (25%)**		**3,212 (69%)**		**1,470 (31%)**		**283 (72%)**		**110 (28%)**		**6,693 (71%)**		**2,668 (29%)**	
**Employment**	**Un-employed**	**Employed**	**Un-employed**	**Employed**	**Un-employed**	**Employed**	**Un-employed**	**Employed**	**Un-employed**	**Employed**	**Un-employed**	**Employed**	**Un-employed**	**Employed**	**Un-employed**	**Employed**
Employment *n (%)*	1,887 (59%)	1,311 (41%)	566 (52%)	522 (48%)	996 (31%)	2,216 (69%)	382 (26%)	1,088 (74%)	110 (39%)	173 (61%)	45 (41%)	65 (59%)	2,993 (45%)	3,700 (55%)	993 (37%)	1,675 (63%)
Acute stress *M (SD)*	42.77 (25.42)		36.84 (25.16)		43.82 (26.16)		35.44 (25.33)		39.31 (28.02)		33.93 (26.48)		43.13 (25.90)		35.95 (25.32)	
Avoidance- sadness *M (SD)*	36.47 (22.32)		32.26 (20.99)		31.67 (22.35)		26.10 (21.00)		28.28 (23.41)		24.94 (22.34)		33.83 (22.53)		28.16 (21.20)	
Distancing-anger *M (SD)*	41.98 (26.29)		38.04 (26.45)		35.80 (25.27)		30.28 (24.73)		30.37 (24.47)		26.96 (25.84)		38.54 (25.96)		33.31 (25.79)	
Generalized anxiety *M (SD)*	48.85 (32.28)		39.65 (31.89)		42.49 (31.84)		31.96 (30.51)		36.99 (31.94)		30.43 (32.37)		45.31 (32.25)		35.03 (31.38)	
Health-related anxiety *M (SD)*	44.98 (31.03)		39.72 (30.64)		43.68 (31.36)		35.72 (30.07)		39.31 (31.96)		31.46 (31.61)		44.12 (31.24)		37.18 (30.44)	
Binge drinking *n (%)*	352 (11%)		180 (16%)		383 (12%)		294 (2%)		48 (17%)		32 (29%)		783 (12%)		506 (19%)	
Emotional abuse *n (%)*	962 (30%)		253 (23%)		860 (27%)		285 (19%)		75 (27%)		28 (25%)		1,897 (28%)		566 (21%)	
Physical abuse *n (%)*	217 (7%)		33 (3%)		159 (5%)		71 (5%)		14 (5%)		8 (7%)		390 (6%)		112 (4%)	
Sex *n (%)*	3,198 (75%)		1,088 (25%)		3,212 (69%)		1,470 (31%)		283 (72%)		110 (28%)		6,693 (71%)		2,668 (29%)	
Lockdown status *n (%)*	4286 (46%)				4682 (50%)				393 (4%)				9361 (100%)			

All respondents were volunteers, which is why the sample is not homogeneous: 57% were employed (5,375), 46% were in complete lockdown (4,286) and did not leave home for any reason, 50% were in partial lockdown (4,682), meaning that they self-isolated but went out for groceries, and 4% were not following any lockdown procedures (393), since they had to go out to work). Ninety-two percentage of the respondents did not report COVID-19 symptoms, (just 8% reported symptoms or suspicions of COVID-19 infection), while 87% said they were living with their relatives. The exclusion criteria had to be a healthcare provider.

Respondents agreed with the terms of our survey regarding privacy and information gathering. The terms of agreement explicitly mentioned that all personal information would be confidential and used to formulate statistical averages. We also informed them that we would use the results for research purposes and publish the findings. We told them that they could choose to opt-out of the study at any time and did not offer financial incentives to answer the survey. Nevertheless, we offered feedback results in which we provided several educational resources regarding COVID-19 and mental health education, such as infographics, videos, and Moodle^®^ online courses. We also informed participants of a hotline where they could receive psychological support if they wished to. The INPRFM Research Ethics Committee approved the study on April 16, 2020. We met the criteria for internet E-surveys such as informed consent, data protection, development, testing, contact mode, advertising the survey, mandatory, voluntary, completion rate, cookies used, IP check, log file analysis, registration, and atypical timestamp considerations (18).

### Instruments

We administered the Questionnaire for the detection of risks to mental health COVID-19 (19–25) online, through a WebApp designed by Linux^®^, PHP^®^, HTML^®^, CSS^®^, and JavaScript^®^, using the latest encryption methods to comply with safety and privacy standards. The survey consisted of four sections: status due to COVID-19, socioeconomic self-report, mental health status, and discrete nominal conditions. The complete questionnaire obtained a Cronbach's alpha of 0.95. A factor loading analysis indicated a common factor for each question above 0.30, with an explained variance of 66.97%.

The self-report on status due to COVID-19 included two questions: What is your current situation? Participants can choose one of six possible answers: I have no COVID-19 symptoms; I have an acute respiratory illness; I suspect I have COVID-19; I have no symptoms, but the person closest to me has COVID-19; I was diagnosed with COVID-19 with no symptoms; I was diagnosed with COVID-19 with symptoms. The second question was: Have you lost someone you love because of the COVID-19? Participants could choose one of seven optional answers: No, I haven't lost anyone; Yes, I lost a daughter or a son; Yes, I lost a wife or a husband; Yes, I lost my mother or father; Yes, I lost a beloved close relative; Yes, I lost my best friend; Yes, I lost my closest colleague. Factor Analysis with the Mineigen criterion 1, iterating criterion 25, Principal Components extraction, Varimax Kaiser Rotation, and correlation Method yielded an explained variance of 54%.

We asked where the person lived, who they live with, their lockdown status, and details about their basic profile on the socio-economic self-report. We asked about their sex, age, marital status, educational attainment, current occupation, current health status, and current physical condition (diabetes, hypertension, obesity, or depression). The self-report on socio-economic status factor loading analysis yielded an explained variance of 52.

**Figure 1:**
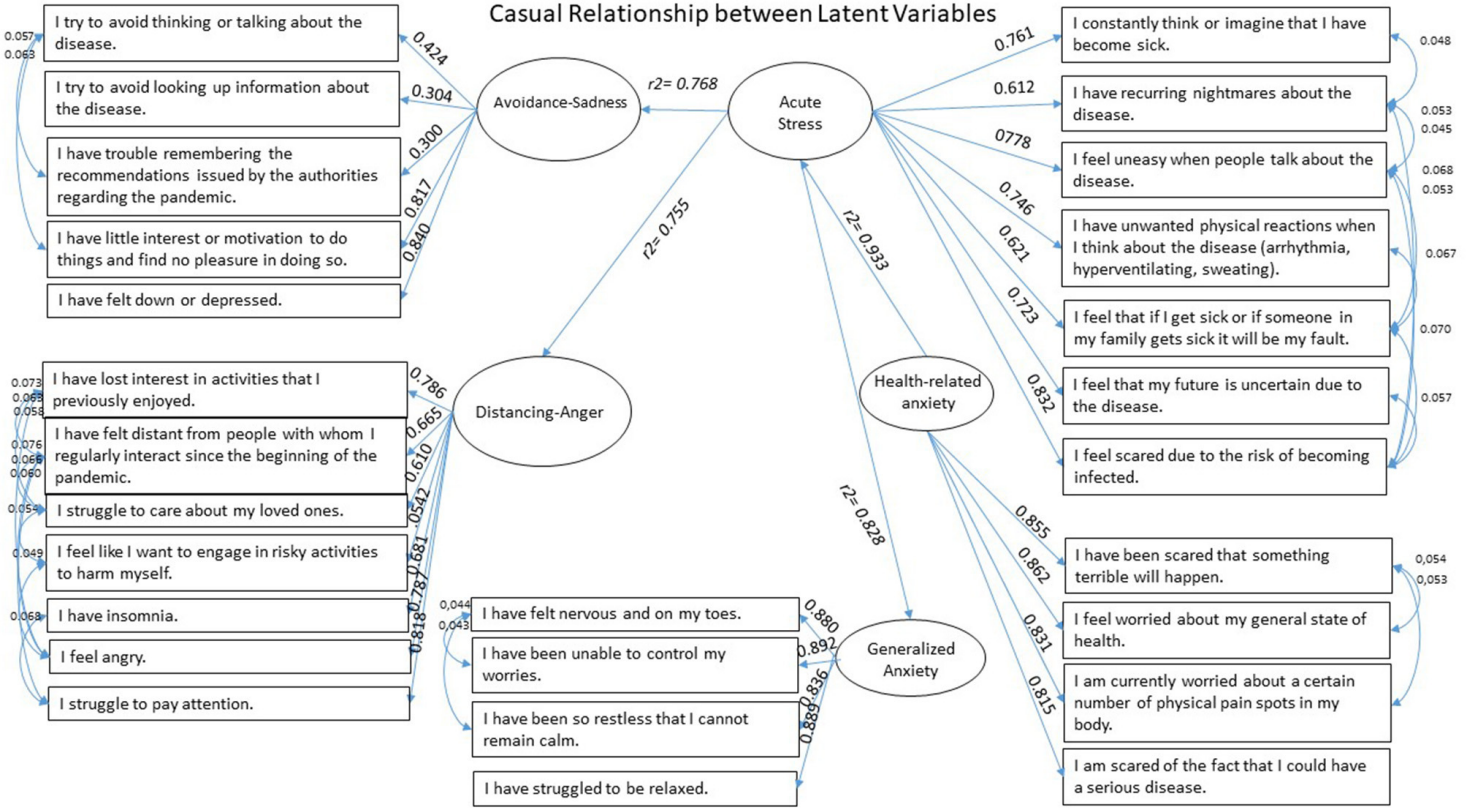
Structural equations model on mental health symptoms: Acute Stress, Avoidance-Sadness, Distancing-Anger, as reported by survey respondents.

We compiled 27 questions to screen for mental health symptoms (see Figure 1). *Acute Stress* is a 7-item subscale referring to the past month, with a Cronbach's alpha of 0.89 and an explained variance of 61%, on which we based on the Post-traumatic Stress Disorder Checklist for DSM-5 (26). Besides, we used the Model Test User Model with Test statistics and obtained factor loadings of between 0.612 and 0.832, with 75 iterations and 29 parameters (*t*_[21]_ = 34,065, *p* = 0.000). We obtained the following index: CFI = 1.000, TLI = 1.000, RMSEA = 0.000 (0.000–0.012), and SRMR = 0.002.

*Avoidance-Sadness* is a 5-item subscale referring to the past month, with a Cronbach's alpha of 0.73 and an explained variance of 72%, taken from the PTSD-Checklist (26). We obtained factor loadings of between 0.300 and 0.840, with 68 iterations and 18 parameters (*t*_[10]_ = 15,913.02, *p* = 0.000). We obtained the following index: CFI = 1.000, TLI = 0.999, RMSEA = 0.014 (0.000–0.028), and SRMR = 0.005.

*Distancing-Anger* is a 7-item subscale with a Cronbach's alpha of 0.88 and an explained variance of 58%, drawn from the 17-item PTSD-Checklist (26) referring to the past month, and the Patient Health Questionnaire (PHQ; 14–16) referring to the past 2 weeks. We obtained factor loadings of between 0.542 and 0.818, with 89 iterations and 30 parameters (*t*_[21]_ = 29,360.57, *p* = 0.001), and the following index: CFI = 1.000, TLI = 1.000, RMSEA = 0.000 (0.000–0.009), SRMR = 0.002.

*Generalized Anxiety* is a 4-item subscale referring to the past 2 weeks, with a Cronbach's alpha of 0.93 and an explained variance of 82%, taken from the field study for the Classification of Mental Disorders for Primary Health Care (ICD-11 PHC; 11). We obtained factor loadings of between 0.836 and 0.889, with 37 iterations and 14 parameters (*t*_[6]_ = 30,032, *p* = 0.000), with the following index: CFI = 1.000, TLI = 1.000, RMSEA = 0.000 (0.000–0.000), and SRMR = 0.000.

*Health-related Anxiety* is a 4-item subscale with a Cronbach's alpha of 0.89 and an explained variance of 76%. We took the first item from the ICD-11 PHC (19) referring to the past 2 weeks, and three items from the Somatic Symptoms without Organic or Medical Cause Current Status Assessment Questionnaire [SSOM; (20, 21)] referring to the present. We obtained factor loadings of between 0.815 and 0.862, with 37 iterations and 14 parameters (*t*_[6]_ = 23,006, *p* = 0.000), with the following index: *CFI* = 1.000, *TLI* = 1.000, *RMSEA* = 0.000 (0.000–0.000), and *SRMR* = 0.000.

The whole 27-item scale used a zero to 10 response scale, in which zero means *absolutely nothing* and 10 means *strongly describes how I feel*,. The Cronbach's alpha from the whole 27-item scale was 0.95, with an explained variance of 62%. So, we summarized the total score of each sub-scale, divided from the maximum possible score, and multiplied by 100 to transform the final qualification into a percentage.

Also, we used three discrete nominal items to explore binge drinking and experience of abuse status. *Binge alcohol consumption* is a question, according to Golberg et al. (19), which determines whether, in the past month, participants have drunk more than five beers, cups, or straight shots, or cocktails with liquor such as tequila, whiskey, mezcal, and rum in <2 h. Participants could answer Yes, I did, No, I didn't, or I rather not answer. We explored the Experience of Abuse with two questions about the past month, based on Morales-Chaine et al. (25). Participants answered whether someone had deliberately harmed them emotionally through verbal humiliation, verbal abuse, physical threats, ignoring them, having restrictions placed on them, or being jealous of them. Participants could answer yes or no. Besides, they reported whether someone intentionally physically hurt them through punches, pushes, pinching, forceful grabbing, or in some other way. Factor loading analysis yielded an explained variance of 52%.

### Data Analysis

We calculated the reliability, validated the subscales, validated the latent variables of the study, and calculated and described the association between them.

Thus, we used the IBM Corporation Released 2010 IBM^®^ SPSS Statistics for Windows, Version 19.0, and the Rstudio^®^ Version. 1.3.959 software.

First, to calculate the reliability and validation of the subscales, we used Cronbach's Alpha and Factor Loading (Mineigen criterion 1, iterate criterion 25, Principal Components extraction, Kaiser Varimax Rotation, and Method correlation) analysis.

Second, to validate the latent variables as mental health symptoms scales, we used Lavaan 0.6–6 to report the iterations number, the confirmatory factor loading analysis, and the structural equation modeling. Specifically, we calculated the Model Optimization Method, the number of free parameters, observations, and missing patterns to validate this model. Furthermore, we used the Model Test User Model with Test statistics, degrees of freedom, *P*-value (Chi-square), and the Model Test Baseline Model. Then, we compared the User Model to the Baseline Model using the Comparative Fit Index (CFI). Moreover, we calculated the Root Mean Square Error of Approximation (RMSEA) with 90% confidence interval-lower—upper ≤ 0.05, the Standardized Root Mean Square Residual (SRMR) their Parameter Estimates with Standard Error Standard and Hessian Observed Information.

Third, we calculated descriptive analysis to describe the average percentage obtained for every mental health symptom. In detail, we computed Means and Standard Deviations.

Fourth, we calculated inferential analysis to describe the mental health average distribution as a function of the lockdown, binge drinking, and experience of abuse. Mainly we computed the Analysis of Variance Type III Sums of Square Sheffe *Post-Hoc* Test and Chi-square test with its *P*-value.

Finally, we used Linear Regression Analysis with the square R using the Enter Method and Durbin residuals procedure to predict relationships between latent variables.

## Findings

In this section, we reported the result of the data analysis. First, we provided the structural equation modeling adjustment ratios to identify the factor loads within each latent variable question and the predictive relationship between variables. Secondly, we described the participants' average scores, according to the threat of mental health problems related to lockdown, binge-drinking, and experience of abuse.

Figure 1 indicates the relationship between each latent variable and its item factor load, obtained through structural modeling equations, derived from 179 iterations and 207 parameters (*t*_[1171]_ = 28,079.418, *p* < 0.001), CFI = 0.947, TLC = 0.940, RMSEA = 0.049 (0.049–0.050), SRMR = 0.048. Results indicate that higher Acute Stress was associated to higher Avoidance-sadness, distancing-anger, and general anxiety (*R*^2^ = 0.768; *R*^2^ = 0.755; and *R*^2^ = 0.828, respectively), while higher health-related anxiety was associated to higher Acute Stress (*R*^2^ = 0.933).

Consequently, in Table 2, we reported participants' mental health average percentages and their lockdown status. On this aspect, the variance analysis (ANOVA) indicates that there were statistically significant differences for acute stress, avoidance-sadness, distancing-anger, generalized anxiety, and anxiety related to physical health levels due to respondents' lockdown status. In the same table, we indicated with an asterisk where were the significant differences between groups. One asterisk indicated if the difference were between *in lockdown* and *in partial* or *not in lockdown* groups. Two asterisks indicated the difference between in partial lockdown group and the not-in lockdown group. For example, Table 2 shows the generalized anxiety levels when people reported being in lockdown (M = 46.52; SD = 32.43), partial lockdown (M = 39.19, SD = 31.80) or not being in lockdown (M = 35.14, SD = 32.16; *F*_[2, 9436]_ = 6.41, *p* = 0.036). Table 2 indicates how many participants reported binge drinking or the experience of abuse according to lockdown conditions (the Chi-squared resulted significative). Twenty-six percentage of the sample experienced emotional abuse (significative differences between groups). Five percentage reported physical abuse (without significant differences between groups).

**Table 2:** Number of respondents by lockdown status during the COVID-19 pandemic and their clinical characteristics.

	**Lockdown/Sex**				
	**In lockdown**	**In partial lockdown**	**Not in lockdown**	***F*_**(2, 9, 436)**_ = *p < * 0.036**	**Total**
Acute stress *M (SD)*	41.27 (25.49)^*^	41.19 (26.19)^**^	37.79 (27.67)^*^,^**^	3.33	41.09 (25.94)
Avoidance/Sadness *M (SD)*	35.15 (22.11)^*^	29.92 (22.09)^*^	27.34 (23.14)^*^	73	32.21 (22.31)
Distancing/Anger *M (SD)*	40.98 (26.38)^*^	34.07 (25.23)^*^	29.41 (24.88)^*^,^**^	99.52	37.05 (26.02)
Generalized anxiety *M (SD)*	46.52 (32.43)^*^	39.19 (31.80)^*^	35.14 (32.16)^*^	69.41	42.39 (32.34)
Health related anxiety *M (SD)*	43.65 (31.01)^*^	41.18 (31.17)^*^	37.10 (32.02)^*^,^**^	12.49	42.14 (31.17)
Binge drinking *n (%)*	532 (12%)^*^	677 (14%)^*^	80 (20%)^*^,^**^		1,289 (14%)
Emotional abuse *n (%)*	1,215 (28%)^*^	1,145 (24%)^*^	103 (26%)^**^		2,463 (26%)
Physical abuse *n (%)*	250 (6%)	230 (5%)	22 (6%)		502 (5%)
Lockdown status *n (%)*	4,286 (46%)	4,682 (50%)	393 (4%)		9,361 (100%)

*A Sheffe Post-hoc Analysis indicated significant differences between*

* *In lockdown and In partial or Not in lockdown groups, and*

** *between In partial Lockdown and Not in lockdown groups. Chi-Square also revealed significant distribution differences between groups*.

Table 3 shows the variance analysis results indicating statistically significant differences between participants' mental health symptoms regarding their binge drinking and abuse experience. For example, people who reported binge drinking had higher levels of acute stress (M = 47.92, SD = 25.62) than those who did not engage in this type of drinking (M = 39.98, SD = 25.78; *F*_[2, 9, 436]_ = 56.45, *p* < 0.001). Notice we indicated with an asterisk where were the significant differences between groups. One asterisk indicated if the difference were between *Yes, I did* group and *No, I didn't* group, or *I would prefer not to answer* group. Two asterisks indicated the difference between *No, I didn't* group, and *I would prefer not to answer* group. It is worth mentioning that those who chose not to answer (*n* = 62, 1%) reported higher average stress levels (M = 52.30, SD = 28.79) than the first two groups mentioned previously. One statistical key difference concerns generalized anxiety (*F*_[2, 9, 436]_ = 79.48, *p* < 0.001). People who binge drank also had higher average generalized anxiety scores (M = 52.51, SD = 32.40) than those who did not (M = 40.74, SD = 32.01; *F*_[2, 9, 436]_ = 79.48, *p* < 0.001). More importantly, regarding physical health anxiety, those who engaged in binge drinking had higher physical health anxiety levels (M = 49.57, SD = 30.47) than those who did not (M = 40.92, SD = 31.09; *F*_[2, 9, 436]_ = 47.73, *p* < 0.001).

**Table 3:** Mental health symptom averages regarding binge drinking, and experience of abuse status during the COVID-19 pandemic, and clinical characteristics.

	**Binge drinking**			
**Mental health symptoms average**	**Yes, I did *M (SD)***	**No, I didn't *M (SD)***	**I would prefer not to answer *M (SD)***	***F*_**(2, 9, 436)**_ = *p < * 0.001**
***N*** **=** **9,361 (100%)**	**1,289 (14%)**	**8,010 (85%)**	**62 (1%)**	**56.45**
Acute stress *M (SD)*	47.92 (25.62)^*^	39.98 (25.78)^*^,^**^	52.30 (28.79)^**^	56.45
Avoidance/Sadness *M (SD)*	39.42 (22.33)^*^	31.01 (22.04)^*^,^**^	47.97 (24.65)^*^,^**^	92.95
Distancing/Anger *M (SD)*	47.73 (26.40)^*^	35.28 (25.47)^*^,^**^	58.57 (30.56)^*^,^**^	147.61
Generalized anxiety *M (SD)*	52.51 (32.40)^*^	40.74 (32.01)^*^,^**^	58.51 (33.52)^**^	79.48
Health-related anxiety *M (SD)*	49.57 (30.47)^*^	40.92 (31.09)^*^,^**^	56.17 (32.82)^**^	47.73
	**Experience of emotional abuse**			
**Mental health symptoms average**	**Yes**, ***M (SD)***	**No** ***M (SD)***	***F***_**(2, 9, 436)**_ **=** ***p<*** **0.001**	
***n*** **=** **9,361 (100%)**	**2,463 (26%)**	**6,898 (74%)**		
Acute stress *M (SD)*	49.16 (24.90)	38.42 (25.74)	311.53	
Avoidance/Sadness *M (SD)*	41.78 (21.76)	29.06 (21.58)	609.13	
Distancing/Anger *M (SD)*	51.81 (25.58)	32.18 (24.27)	1120.6	
Generalized anxiety *M (SD)*	57.51 (30.58)	37.40 (31.34)	733.56	
Health-related anxiety *M (SD)*	52.60 (29.94)	38.70 (30.80)	363.42	
	**Physical abuse experience**			
**Mental health symptoms Average**	**Yes**, ***M (SD)***	**No** ***M (SD)***	***F***_**(2, 9, 436)**_ **=** ***p<*** **0.001**	
***n*** **=** **9,361 (100%)**	**502 (5%)**	**8,859 (95%)**		
Acute stress *M (SD)*	47.82 (26.48)	40.75 (25.87)	32.36	
Avoidance/Sadness *M (SD)*	42.71 (22.81)	31.68 (22.15)	107.14	
Distancing/Anger *M (SD)*	53.15 (26.82)	36.23 (25.71)	187.08	
Generalized anxiety *M (SD)*	57.77 (31.23)	41.60 (32.20)	109.78	
Health-related anxiety *M (SD)*	52.08 (29.73)	41.64 (31.16)	48.91	

*A Sheffe Post-hoc Analysis indicated significant differences between*

* *Yes, I did and No, I didn't or I would prefer not to answer groups, and*

** *No, I didn't or I would prefer not to answer groups*.

Regarding the report on victims of emotional abuse, variance analysis indicated statistically significant differences between all mental health symptom averages. For example, Table 3 shows that participants reporting the experience of emotional abuse had higher average levels of distancing/anger (M = 51.81, SD = 25.58) than those who did not (M = 32.18, SD = 24.27; *F*_[2, 9, 436]_ = 1120.60, *p* < 0.001). As for general anxiety and emotional abuse, victims also had higher average scores (M = 57.51, SD = 30.58) than those who had not been victims of this (M = 37.40, SD = 31.34; (*F*_[2, 9, 436]_ = 733.56, *p* < 0.001).

Regarding the experience of physical abuse, variance analysis also revealed statistically significant differences between all the mental health symptom averages. Those who reported this type of abuse had higher average levels of acute stress (M = 47.82, SD = 26.48) than those who did not (M = 40.75, SD = 25.87; *F*_[2, 9, 436]_ = 32.36, *p* < 0.001; Table 3). Finally, those who had suffered physical abuse had higher general anxiety average scores (M = 57.77, SD = 31.23) than those who did not (M = 41.60, SD = 32.20; (*F*_[2, 9, 436]_ = 109.78, *p* < 0.001).

## Discussion

The purpose of this paper was to describe the relationship between mental health symptoms, binge drinking, and experience of abuse during the lockdown. First, we describe the acute stress levels and their relationship with other mental health symptoms. We found that health-related anxiety was associated with acute stress symptoms and high avoidance, sadness, distancing, anger, and anxiety during the COVID-19 pandemic. It appeared that total lockdown was associated with higher levels of mental health symptoms compared with partially or not been in confinement. Specifically, we observed that acute stress symptoms were higher in complete lockdown and partial lockdown than when people aren't in lockdown. Also, generalized anxiety symptoms were similar in partial and when people aren't in confinement, being both lower than when people reported total lockdown. Otherwise, we found a high proportion of people who reported binge drinking when they were in partial lockdown followed by those who were in total confinement and those who were not in lockdown. We observed higher emotional abuse when people said to be lockdown than partial or not in confinement status. Levels of physical abuse experience were similar for everyone, though. Finally, we also found high acute stress, avoidance, sadness, distancing, anger, and anxiety symptoms when people reported binge drinking, emotional, or physical abuse experiences.

Our participants' acute stress symptoms were similar to those found by Rogers et al. (4). Specifically, we found constant thinking of becoming sick, recurring nightmares, unwanted physical reactions when thinking about the disease, fault about others getting sick, uncertain future expectations, and feeling scare due to the risk of becoming infected. Findings agree with insomnia, anxiety, irritability, and sleep disorder reported by Rogers et al. (4). Moreover, the acute stress symptoms were also like those evaluated by Blevins et al. (26) when threatening events occur. We found similar mental health symptoms as those observed during and in the aftermath of other traumatic events, such as the Ebola epidemic (4).

According to De Camargo (9) and Selye (8), people, in general, can respond to a traumatic or stressful event through a general adaptation syndrome, composed of an awake phase, a resistance phase, and an exhaustion phase. Some of our participants could be an on awake stage which is akin to being in a state of alert, but it also includes a moment of freezing in response to the stressful event. High-stress levels allow systems to be active in the resistance phase for weeks or months, if not years. Some of the participants can be experiencing this second phase of stress. In the exhaustion phase of stressful events as the pandemic COVID-19, people report tiredness that is usually persistent. This third phase is when the following reactions occur: insomnia, fatigue, lack of concentration, but cardiovascular and metabolic reactions, endocrine responses, emotional problems, gastrointestinal issues, and vascular events, among others (5, 6, 10, 11). Consequently, our findings suggested that 1 month into lockdown during later stages of the pandemic COVID-19 was associated with the resistance phase symptoms characterized by acute stress reactions; these were higher in lockdown status than been not in lockdown during the later stage of the COVID-19 pandemic in Mexico. Results are just like the ones reported by Ho et al. (13).

Additionally, findings indicated that health-related anxiety symptoms were associated with acute stress symptoms. Health-related anxiety symptoms are those where people report been scared that something terrible could happen, feel worried about the general state of health, worried about physical pain spots in the body, or of the fact that people could have the severe disease (20, 21). Consequently, health-related anxiety looks to associate with acute stress as the traumatic event to be solved (26). In this sense, results also indicated that acute stress was associated with avoidance, sadness, distancing, anger, and anxiety symptoms, just as Zhue et al. (27) reported. Blevins et al. (26) described avoidance symptoms similarly to ours; it means we assessed avoiding thinking and talking about the disease, avoiding looking up information about it, and having trouble remembering the pandemic's recommendations. Therefore, acute stress accounts for the avoidance symptoms during the pandemic COVID-19 in this sample.

Moreover, sadness symptoms were those by Arrieta et al. (22), Arroll et al. (23), and Mitchell et al. (24) suggested. It means sadness consists of having little interest or motivation to do things, finding no pleasure in doing so, or feeling down or depressed. This sadness was also associated with acute stress. Such results are like those reported by Li et al. (12), Morales-Chainé et al. (15), and Rogers et al. (4), probably correlated with the pandemic COVID-19. Acute stress was also related to conditions of distancing and anger reports. It consisted of circumstances such as losing interest, becoming distant from others, struggling to be empathetic, wishing to self-harm, having insomnia, feeling anger, and struggling to pay attention. Distancing and anger symptoms were the ones De Camargo (9), Morales-Chaine et al. (15), Rogers et al. (4), and Selye (8) also suggested happens in difficult situations or events. Additionally, we found that the acute stress related to anxiety, defined as feeling nervousness, unable to control concerns, uneasiness, and difficulty relaxing. Goldberg et al. (19) and Li et al. (7) reported these kinds of symptoms due to the event or the pandemic COVID-19, respectively.

Likewise, our findings suggested lockdown has some effects on mental health symptoms. As we already established, acute stress symptoms were higher when people reported total and partial lockdown than when they were not in confinement at all. Consequently, we need to explore how full or partial lockdown works over the body to relate as a predictor of acute stress. Although Rogers et al. (4) and Morales-Chaine et al. (15) reported the possible role of pandemic over the mental health symptoms, our results indicated the critical role of these two kinds of lockdown, complete and partial, not just over acute stress but also over the other mental health symptoms. Specifically, we found that the type of lockdown affected differentially people who reported different levels of avoidance, sadness, distancing, anger, and both kind of anxiety. Levels were higher in complete lockdown followed by partially and not in lockdown conditions with the lower risk levels. Notice that findings suggested that generalized anxiety symptoms were similar when people reported partial or not confinement, both lower than those who reported total lockdown status. Then, anxiety is high at complete confinement, as Li et al. (12) and Ho et al. (13) wrote.

Otherwise, we found a high proportion of people who reported binge drinking when they were in partial lockdown followed by complete confinement and not in lockdown at all. However, men and women who said to consume more than five drinks in <2 h reported higher levels of acute stress, avoidance, sadness, distancing, anger, and anxiety than those who didn't drink that way. Even though these results doesn't mean a causal relationship, they are like Ho et al. (13) reported. It seems people are fearful of pandemic COVID-19 and that a risk of contagion creates anxiety levels associated with alcohol binge drinking. Furthermore, we observed that participants who deliberately were harmed emotionally, through verbal humiliation, verbal abuse, physical threats, being ignored, having restrictions placed on them, or being jealous of them, had high mental health symptoms. Meanwhile, participants who reported someone physically hurt them through punches, pushes, pinching, or forceful grabbing had high mental health symptoms.

In general, findings suggested that being in lockdown during the COVID-19 pandemic has affected a large sector of the population's psychological state. People have reported feelings of stress explained by health-related anxiety and other mental health symptoms, such as avoidance, sadness, distancing, anger, and generalized anxiety. Mental health symptoms became severe when complete lockdown, binge alcohol drinking, or experience abuse.

Li et al. have reported all mental health symptoms we explored here during situations in which there is vicarious trauma (2020). As well, these findings match those found by Mortensen and collaborators (28), who also reported negative thoughts (such as anxiety), and Li et al. (12), whose study found negative cognitive situations among respondents (mainly dominated by anxiety and profound sadness), during the early weeks of COVID-19 lockdowns. Also, and regarding binge drinking, it is essential to highlight the role of this behavior could be playing an exacerbating mental health symptom (13, 19, 27), as noted in The Lancet (3). All the findings corresponded to Selye (8), Blevins et al. (26), and De Camargo criteria to predict Post-Traumatic Stress Disorder (2010). Then, further studies should analyze the evolution of these conditions up to the exhaustion stage, which could constitute a PTSD condition.

Nevertheless, the results we have been discussing here seems relevant, we need to consider that the stress levels and mental health symptoms found during this study could also be a reaction to other social determinants that future research must address. Further efforts should consider the role that friends, and family networks play in regulating the effects of acute stress caused by lockdowns or violence (12). We should analyze the relationship between these variables, given that the results found in this paper refer to participants who were in confinement with their families, which might some way have been an attenuating factor.

However, our findings address early risk detection strategies, allowing us to prevent high morbidity situations associated with emotions, cognition, and mechanisms whereby people deal with life-threatening conditions. It hence the importance of developing strategies based on empirical data (29), intended to intervene in the most appropriate way to reduce the risk of stress, anxiety, and sadness, using remote methods, as recommended by Zhue et al. (27). It is essential to conduct proper research tailored to each community to deliver the most effective response. Consequently, this paper provides a tool for the early detection of health risks derived from stress, anxiety, and sadness during the COVID-19 pandemic, with an approach based on the use of Smartphones that will allow us to identify mental health symptoms while people are in lockdown. It will supply the data needed to act accordingly (30). We, therefore, recommend remote psychological care (27), based on scientific evidence, to cope with emotional, cognitive, and behavioral attitudes toward loss, acute stress, negative thoughts, emotions, anxiety, alcohol consumption, and the experience of abuse.

Because of our findings, we considered important Li et al. (7) concerns. It means that the levels of vicarious trauma could be effectively handled through specific prevention guidelines if correctly adopted by the authorities. Then, it is essential to note that decision-making at the government can enhance or diminish citizens' physical and psychological health (7). Authorities could have an impact on reducing stress and anxiety levels. We would recommend prevention policies and campaigns to promote mental health at the national level to reduce stress levels, avoidance, sadness, distancing, anger, anxiety, substance use, and abuse experience during and after the COVID-19 pandemic. We could achieve it through a series of systematic multi- and inter-disciplinary systematic strategies at all levels of care to make the best use of the human and economic resources in Mexico.

Further studies should contribute to early risk detection strategies and ways to promote remote psychological education to improve mental health by understanding the different illnesses, mechanisms of action, and evolution. It is also necessary to consider the timeframe in which we measure mental health symptoms since reaction times vary on each country's social conditions and respondents' current mental health status.

## Limitations

Even though the current study made it possible to describe the mental health symptoms during the lockdown, it has several limitations. One suggestion for overcoming this involves using a cross-sectional design. This study doesn't allow us to identify precedent social or health conditions to the pandemic COVID-19. Social determinants before and during lockdown represent crucial factors to explore and relate to other actual needs like near-deaths or losing jobs. A cross-sectional study takes a one-shot picture that opens a lot of research needs we didn't explore. One of the crucial factors could be previous mental health conditions that could worsen through pandemic COVID-19, events such as getting sick or converting oneself on primary take giver. Consequently, additional studies should evaluate, diagnose, and especially follow up on mental health conditions and their evolution during and after the COVID-19 pandemic. Although it is essential to detect early mental health risks, there is a need for procedures that will make it possible to identify symptoms and their social determinants to interrupt their progression or develop illness effectively. We need to address studies exploring mechanisms and causal relationship between mental health symptoms and binge drinking or abuse experiences or lockdown conditions.

Although an online survey has its advantages, it also has certain limitations as the biases resulting from the necessary technical requirements. Therefore, the findings are not generalizable to the whole population, and it is essential to evaluate the robustness of the survey for diagnosing mental disorders or risk levels. We need to keep looking for signs as an early screening and self-awareness procedure recommended by the Pan-American Health Organization to cease their progression, (i). It is essential to enhance the understanding of the situation and promote copying actions focused on reducing the symptoms and diagnosing disorders reducing the treatment gap. Accordingly, subsequent studies should conduct a follow-up assessment of the symptoms addressed in this paper and prioritizing research on the role of acute stress over other mental health conditions.

Another recommendation concerns the exclusion criteria limiting respondents of those over 16. Even though we should include a broader age group to study mental health symptoms, subsequent studies should consider exclusion criteria where just people older than 16 ensuring control age comprehension factors. Our final recommendation is to include social determinants as control variables. This study failed to measure the role of employment and how it contributes to the early emergence of mental health symptoms, binge drinking, or the experience of abuse. Future research should consider studying how social determinants regulate the emergence of mental health symptoms.

## Data Availability Statement

The original contributions presented in the study are included in the article/supplementary material, further inquiries can be directed to the corresponding author/s.

## Ethics Statement

The studies involving human participants were reviewed and approved by Ethics Committee of the Instituto Nacional de Psiquiatría Ramón de la Fuente Muñiz. The patients/participants provided their written informed consent to participate in this study.

## Author Contributions

MR contribuyó a la redacción y análisis inicial de datos. SM, AL, ABo, ABe, RR, CG, and CA contribuyeron a la revisión y revisión del análisis and interpretación y discusión de datos. All authors contributed to the article and approved the submitted version.

## Conflict of Interest

The authors declare that the research was conducted in the absence of any commercial or financial relationships that could be construed as a potential conflict of interest.
